# Ethical decision-making for large language model use: a six-step model for mental health professionals

**DOI:** 10.3389/fpsyg.2026.1998626

**Published:** 2026-10-20

**Authors:** Russell Fulmer, Yiying Xiong, Yusen Zhai, Jungeun Lee, Gustav Nkya, Robert Naugle

**Affiliations:** 1 Department of Special Education, Counseling, and Student Affairs, Kansas State University, Manhattan, KS, United States; 2 School of Education, Johns Hopkins University, Baltimore, MD, United States; 3 School of Human Development, University of Florida, Gainesville, FL, United States; 4 Psychological, Health, and Learning, University of Houston, Houston, TX, United States; 5 Kilimanjaro Clinical Research Institute, Moshi Urban, Tanzania

**Keywords:** artificial intelligence (AI), deceptive empathy, digital attachment, ethical decision-making model, large language models (LLMs)

## Abstract

This article presents a six-step ethical decision-making model for mental health professionals confronted with ethical concerns arising when clients use large language models (LLMs) for mental health support. LLMs may introduce ethical problems that existing decision-making models were not designed to address. To provide guidance, we synthesized two bodies of research, the first concerning ethical risks posed by LLM use, and the second about existing decision-making models, to create a new model designed specifically for LLM and AI-related ethical issues. Our model addresses AI-specific phenomena such as deceptive empathy, algorithmic bias, digital attachment, and diffuse accountability structures. We propose this model as a conceptual foundation and encourage its ongoing refinement as AI continues to evolve.

## Introduction

The emergence of artificial intelligence (AI) has created new ethical issues and dilemmas for mental health professionals who may lack the necessary guidance to address them. Clients increasingly use generative AI and large language model (LLM) tools such as ChatGPT for mental health support, information, and guidance. Although the ethical concerns are relevant across mental health professions, the present model focuses on situations in which a mental health professional learns about a client’s use of an LLM and must consider its implications for the client’s well-being and the professional relationship. The model does not address professionals’ own use of LLMs in clinical practice.

Mental health professionals may turn to several sources for ethical guidance. The first is the American Counseling Association (2024), which issued a series of recommendations for implementing AI across clinical use (broadly construed), assessment and diagnosis, and counselor education. Other associations followed suit and issued guidelines for the use of AI. Second, counselors may extrapolate from existing ethics codes, which are often general in their treatment of technology and do not focus specifically on AI. The ACA is updating its 2014 code, and AI-specific guidance may or may not be included. Third, counselors may extrapolate from existing models of ethical decision-making. Many models exist across counseling and related mental health professions, with distinct foci and underlying philosophies. A prominent example is the ethical bracketing strategy proposed by Kocet and Herlihy (2014) to deal with value conflicts.

The fourth source of guidance is the expanding body of literature on AI and counseling specialties, including counselor education (Mifsud and Herlihy, 2026; Gonzalez, 2025; Han and Shin, 2025; Maurya and Cavanaugh, 2025; Sheperis et al., 2025; Shoemaker et al., 2025; Brue and Brue, 2024; Fulmer, 2023). Research also pairs AI with the counseling curriculum implied by the Council for the Accreditation of Counseling and Related Educational Programs (CACREP), such as lifespan development (Fulmer and Zhai, 2025), research and program evaluation (Gantt-Howrey et al., 2025; Watson et al., 2025; Shaw et al., 2023), assessment and diagnostic processes (Beeson et al., 2025; Rensi et al., 2025), and social and cultural identities and experiences (Day, 2023), each offering ethical direction and insight for AI application in particular contexts. Collectively, this literature provides important ethical guidance for AI use in counseling. Yet, guidance specifically addressing professionals’ ethical responses to clients’ own use of LLMs remains limited. The fifth source of guidance is for counselors to seek advice by consulting with colleagues or utilizing professional resources, such as the ACA’s ethics consultation service. The current sources available for guidance represent a helpful (e.g., Step 3 of our model is “Consultation”), yet incomplete and disjointed landscape for AI ethical decision-making.

## LLM ethical risks

People seeking mental health support may use LLMs (Stade et al., 2024). There is evidence of ethical risks associated with the use of LLMs in mental health contexts (Iftikhar et al., 2025; Moore et al., 2025). Mental health professionals often cite privacy, confidentiality, safety and harm, and the dehumanization of counseling as concerns of using LLMs or AI clinically (Meadi et al., 2025; Gorelik et al., 2025). Moore et al. (2025) found that LLMs may perpetuate mental health stigma toward those with certain mental health conditions. Iftikhar et al. (2025) conducted an 18-month ethnographic study with mental health practitioners. Their qualitative analysis included 137 sessions: 110 self-counseling sessions conducted by peer counselors and 27 simulated sessions evaluated by clinical psychologists. The analysis identified 15 ethical violations across five major themes: lack of contextual understanding (oversimplified, one-size-fits-all interventions), poor therapeutic collaboration (limited user agency), deceptive empathy (false emotional connection through anthropomorphic responses), unfair discrimination (algorithmic bias toward marginalized populations), and inadequate safety and crisis management (differential harm based on users’ clinical knowledge and digital literacy). It should be noted that the researchers primarily collaborated with psychologists rather than counselors for the study. Their conclusions were derived from data from a single online mental health support platform that delivers CBT-based interventions. Also, as the authors noted, the LLMs were asked to perform as if they were mental health clinicians, but of course, they are not actually counselors. In summary, there is growing evidence of ethical breaches, violations, and clinician worry regarding the use of LLMs in practice.

## Existing ethical decision-making models

Ethical decision-making guidance specific to AI has begun to emerge. For example, Pillay (2025) proposed AI-related ethical decision-making guidelines for mental health clinicians. However, the present model has a different focus: ethical concerns that arise when clients independently use LLMs for mental health support, information, or guidance. Many decision-making models are grounded in differing theoretical foundations, including social constructivism (Cottone, 2001), hermeneutic theory (Clark, 2012), and value theory (Kocet and Herlihy, 2014). Forester-Miller and Davis (2016) offer a widely used and pragmatic approach. Their model synthesizes five previous ethical decision-making frameworks and incorporates the foundational ethical principles of autonomy, justice, beneficence, non-maleficence, and fidelity into a seven-step process. Their integrative approach made a significant contribution to the ethical decision-making literature.

Johnson et al. (2022) conducted a review of 1,520 articles pertaining to ethical decision-making, including the work of Forester-Miller and Davis. Their analysis identified 38 distinct ethical decision-making models. Each model was coded to identify discrete components. For example, one component found in many models is “prediction,” which entails considering the consequences of a chosen action, including estimating the probability of expected outcomes and identifying potential ethical traps associated with the intended course of action. The researchers found substantial overlap across models. For instance, “action and deliberation” appeared in 92% of the models reviewed, while even the least frequently represented component, the “intrapersonal,” appeared in 45% of models. From this analysis, a taxonomy was constructed consisting of eight ethical decision-making components. In sum, the taxonomy identified, described, and interpreted the core elements common across multiple ethical decision-making models (Johnson et al., 2022). The present research team synthesized and reorganized the overlapping components identified in Johnson et al.’s (2022) taxonomy into a six-step model intended to address ethical concerns arising when clients use LLMs for mental health support, information, or guidance. Rather than mapping each component to a single step, we combined related components to create a practical framework that retains the central processes of ethical decision-making while addressing concerns specific to client use of LLMs. Table 1 illustrates how the eight components relate to the six steps.

**Table 1:** Relationship between Johnson’s components and present six-step model.

Johnson component	Related step(s) in our six-step model	Rationale
Action	Step 4: action	Both involve implementing a selected course of action.
Review	Step 1: problem recognition and interpretation; Step 3: consultation	Problem clarification and professional consultation are supported through gathering relevant information and reviewing applicable ethical standards
Intrapersonal	Step 6: reflection and prevention	Examining one’s own assumptions and reactions supports reflection.
Interpersonal	Step 3: consultation; Step 4: action	Consultation involves communication and deliberation, while action may involve informing affected parties.
Prediction	Step 2: risk assessment; Step 5: monitoring consequences	Assessing potential harm and anticipating outcomes supports risk assessment and ongoing monitoring.
Deliberation	Step 1: problem recognition and interpretation; Step 3: consultation	Defining the problem and evaluating alternatives are central to interpretation and consultation.
Self-monitoring	Step 5: monitoring consequences; Step 6: reflection and prevention	Evaluating outcomes and revisiting decisions support ongoing monitoring and reflection.
Perspective-taking	Step 1: problem recognition and interpretation; Step 3: consultation	Understanding the client’s circumstances and considering the perspectives of affected parties inform interpretation and consultation.

The mappings are conceptual rather than exclusive. Several components inform more than one step, and the steps may overlap.

## Six-step ethical decision-making model

Our model is designed as a flexible guide rather than a rigid protocol. The steps may overlap. Practitioners are encouraged to use their clinical judgment and, depending on the specific ethical context, modify the guidelines accordingly. The case example presented later illustrates this modification in practice.

Our model is grounded in procedural and relational ethics and informed by the ACA’s foundational ethical principles of autonomy, non-maleficence, beneficence, justice, fidelity, and veracity. These principles guide mental health professionals in their decision-making when addressing ethical concerns related to LLM use. Although the ACA Code of Ethics informs the development of our model, professionals in related mental health disciplines should consult the ethical codes and requirements applicable to their own professions. Our model is intended to provide a common decision-making structure rather than to replace discipline-specific ethical standards.

Iftikhar et al.’s (2025) five themes informed our model by identifying potential concerns when clients use LLMs for mental health support. Because the Iftikhar study examined LLMs acting in a counseling role, these themes should serve as prompts for inquiry rather than evidence that ethical violations have occurred in client-use situations. Practitioners should explore how their clients perceive LLM use as beneficial and identify any potential concerns.

The following sections summarize and elaborate upon the components of our six-step model.

1. *Problem recognition and interpretation* involves defining and evaluating aspects of the situation, identifying available options, and clarifying the ideal or ethically preferred outcome.
2. *Risk assessment* is an ongoing process centered on evaluating the severity of potential client harm and appraising the likelihood of subsequent or escalating harm.
3. *Consultation* involves communication among relevant parties and collaborative deliberation toward ethical consensus.
4. *Action* involves informing affected parties of the decision and may include advocacy as part of implementation.
5. *Monitoring consequences* involves documenting outcomes, evaluating the effects of the decision, and reengaging with the ethical decision-making process if necessary.
6. *Reflection and prevention* involves examining both the case and the counselor’s own reactions, assumptions, and emotional responses while identifying strategies to prevent future AI-related ethical breaches.

To ensure clarity, definitions of the technologies addressed in our model is important. AI is an umbrella term for a range of computational techniques that exhibit human-level cognitive and problem-solving abilities (Fulmer et al., 2025; Shaw et al., 2023). Within this field, LLMs are a subset trained on extensive text corpora to generate human-like language. Relatedly, Generative Pre-trained Transformers (GPTs) are a specific family of LLMs built on the Transformer architecture (Langlais et al., 2025). Thus, when counselors use ChatGPT, they are interacting with a GPT-based LLM, under the wider AI umbrella. For simplicity, the remainder of this article uses the terms AI and LLM interchangeably to refer broadly to systems relying on large language model technology within the AI-domain.

### Step 1: problem recognition and interpretation

Definition: Describe the ethical concern and explain the nature of the problem within its context. Identify whether the ethical issue arises from the outputs of an LLM. Examine who bears responsibility for the concern, recognizing that accountability may rest with the counselor, the AI developer, the organization deploying the technology, or other stakeholders. Although accountability may be distributed, counselors remain responsible for their own professional decisions and for responding to concerns about client welfare within their ethical and legal obligations.

Iftikhar et al.’s (2025) five themes can be adapted as domains of inquiry when counselors assess their clients’ LLM use. In this context, the themes are not presumed ethical violations. Rather, they identify areas counselors may explore for possible harm, such as perceived empathy or discrimination. The themes are summarized below.

1. *Lack of Contextual Understanding:* Does the client report generic advice disconnected from their cultural background, identity, or lived experience? Be alert to oversimplified responses to complex trauma.
2. *Poor Therapeutic Collaboration:* Does the LLM dominate conversations with lengthy monologs? The client may be placed in a passive role or disengaged, and their own insights may be dismissed or overridden.
3. *Deceptive Empathy:* The LLM may use formulaic emotional language (“I hear you,” “I understand”), create a sense of false understanding, or use self-referential statements.
4. *Unfair Discrimination*: The LLM may make stereotypical assumptions about the client based on their demographics, reinforcing stereotypes and potentially harming marginalized groups (Ranjan et al., 2024). Be alert for culturally insensitive recommendations.
5. *Inadequate Safety and Crisis Management:* LLMs may respond inappropriately to suicidal ideation, fail to escalate crisis situations to a human being, or abruptly disengage when clients raise topics the LLM perceives as unsuitable.

### Recommendations

Because ethical concerns or violations may emerge at any point in counseling, practitioners should continuously assess for LLM-related risks using clinical judgment. Counselors may need to proactively inquire about clients’ use of LLMs because clients may not disclose such use or recognize when ethical concerns arise. Particular caution may be warranted with vulnerable populations, as research has documented dangerous LLM responses to suicidal ideation (Hill, 2025; Shah et al., 2025). Counselors should also remain attentive to the possibility that clients may anthropomorphize AI systems and develop reliance or attachment to them because of their human-like and seemingly empathetic responses (Alabed et al., 2022; Timmons et al., 2023). Accordingly, counselors should document their concerns about client–LLM interactions and monitor for signs of attachment or dependence, which emerging research suggests may contribute to loneliness, problematic use, and reduced socialization (Fang et al., 2025; Yang and Oshio, 2025; Xie and Pentina, 2022).

Practitioners must distinguish between a user’s *perceived* empathy or trustworthiness of an LLM and the model’s actual *warranted* trustworthiness. Human-like responses may encourage reliance despite the system’s limited capacity for accountability (Sedlakova et al., 2026; Iwasaki, 2026). Practitioners should also assess privacy and confidentiality concerns associated with client LLM use, including what personal or sensitive information clients share with these systems and whether they understand how their data may be collected, stored, and used. These concerns warrant notable attention with minors, who may have limited understanding of data practices and the implications of sharing sensitive information. When appropriate, practitioners should address LLM use as part of informed consent and discuss relevant privacy limitations with clients and, when applicable, parents or guardians.

### Step 2: risk assessment

Definition: This step is primarily concerned with identifying the degree of harm experienced by the client, if applicable, and assessing the probability of additional harm. Non-maleficence is the overarching ethical principle germane to risk assessment. Although risk assessment should be carried out throughout the therapeutic process, here we dedicate a step entirely to it. We classify ethical risks by their severity level and intervention timeframe. Table 2 classifies LLM ethical issues into three levels, with descriptions and examples. A low risk situation may require broaching the topic with the client. A moderate-risk situation would warrant closer monitoring, continued discussion, and perhaps consultation. Risk is categorized as “high” when there is a serious risk of physical or psychological harm requiring prompt professional intervention. The urgency and nature of the response should depend on factors such as the severity and immediate threat of the potential harm.

**Table 2:** Ethical concern stratification.

Risk level	Description	Examples
Low	Limited evidence of harm and no immediate indication of significant danger. Continued attention and monitoring are appropriate because circumstances may change.	No/mild distress; LLM used for psychoeducation or emotional support with only minor or contextual inaccuracies.
Moderate	Concern about potential harm, with indications that the situation could worsen or affect the client’s well-being or professional relationship.	Reinforcement of distorted thinking; deceptive empathy contributing to false attachment; reinforcement of stigma; oversimplified or contextually disconnected advice.
High	Serious risk of harm to the client or others requiring prompt professional intervention. Situations involving imminent or escalating danger may require immediate crisis intervention.	Suicidal ideation is ignored or reinforced; client intends to follow potentially harmful LLM-generated medical advice; severe discriminatory or otherwise dangerous output.

### Recommendations and warnings

High-risk concerns require prompt professional intervention. When assessment indicates imminent danger or another clinical emergency, crisis intervention protocols should commence immediately. Thoroughly document the incident, including the timeline and the messages leading up to the disclosure or recognized concern. Maintain heightened awareness of risks associated with LLM use among vulnerable populations, including susceptibility to influence and the formation of dependence or attachment (Palaniyappan et al., 2026; Namvarpour et al., 2026). Incidents involving adolescent suicide underscore the imperative to restrict, limit, or closely supervise LLM use among youth (Bakir and McStay, 2025; Hill, 2025).

If a moderate risk is determined, consider discussing with the client the possibility of AI-generated errors, sometimes referred to as “hallucinations,” a term used to designate outputs that are factually incorrect, fabricated, or nonsensical (Maleki et al., 2024; Emsley, 2023). Clients should understand that LLMs are fallible and that their outputs should not be treated as authoritative. Document the concern and monitor for escalation to a higher risk level.

If a low risk is determined, practitioners should broach the topic when appropriate. A low-risk designation should not be treated as a reason to disregard the identified concern. Practitioners should monitor their client’s use and reliance on the LLM. Research suggests that increasing use, cognitive offloading, and replacing human connection with chatbots may be signs of growing dependence on LLMs (Head, 2025).

As a big-picture note on AI and risk assessment, it is worth acknowledging that AI is not uniformly harmful in clinical contexts. The same technology that poses ethical risks may also offer useful clinical tools. Research indicates that AI shows promise in suicide risk assessment through linguistic pattern analysis and risk classification accuracy (Esmaeilzadeh, 2025; Bernert et al., 2020). Counselors are therefore encouraged to resist dichotomizing AI as entirely harmful or entirely beneficial. Its clinical value or risk depends significantly on context, the specific application, the population using it, and the degree of human oversight involved.

### Step 3: consultation

Definition: This step involves consultation with professional codes of ethics, knowledgeable colleagues, and professional services such as ACA ethics consultation. The aim is to clarify professional standards and ethical responsibilities. Practitioners should identify the potential harm or concern arising from the client’s LLM use and determine which ethical principles and professional standards guide their response. Consultation can help clarify the counselor’s responsibilities and appropriate course of action.

The ACA Code of Ethics guides counselors’ professional conduct; it does not govern LLMs. Accordingly, the standards listed in Table 3 are relevant to counselors’ responses to potential concerns arising from clients’ LLM use, not to violations committed by the technology. The examples are illustrative rather than exhaustive. Counselors should consult the full ACA Code and seek guidance when the applicable standard is unclear.

**Table 3:** Potential LLM-related concerns and relevant ACA ethical standards.

Potential concern involving LLM use	Relevant ACA ethical standard	Professional responsibility and response
Lack of contextual understanding	A.1.a. Primary responsibility	Practitioners should attend to the client’s individual circumstances and promote the client’s welfare when evaluating generic or contextually inappropriate LLM responses.
Cultural insensitivity or invalidation	A.1.a. Primary responsibility	Practitioners should assess whether culturally insensitive or invalidating LLM responses have affected the client’s wellbeing.
Deceptive empathy	A.4.a. Avoiding harm	Practitioners should assess whether an LLM’s human-like emotional language has contributed to distress, misunderstanding, or problematic attachment.
Inadequate crisis response	B.2.a. Serious and foreseeable harm and legal requirements	When serious and foreseeable harm is identified and protective action may require disclosure of confidential information, counselors should follow B.2.a requirements, applicable law, and relevant crisis procedures.
Algorithmic bias or discriminatory outputs	C.5. Nondiscrimination	Practitioners should assess potential effects of biased or discriminatory outputs and respond to protect clients from harm and discrimination.

### Recommendations

Many professionals are available for consultation, including supervisors, colleagues, or the ACA professional ethics consultation service. All consultations should be properly documented. The conclusions reached through consultation should directly inform the action taken in Step 4. We advise the counselor to ensure their response is grounded in professional standards and ethical reasoning rather than individual opinion alone.

### Step 4: action

Definition: This is the intervention step, in which counselors take action with client welfare at the forefront. Action should be part of a broader strategy that depends on the nature of the specific ethical breach, takes into consideration guidance obtained through consultation, ethics code review, and risk assessment, and is collaborative with the client whenever possible. Gonzalez and Dollarhide (2025) suggest that AI functions not merely as a clinical tool but as a multi-systemic force affecting individuals, relationships, institutions, cultures, and professional identity over time. Applying Bronfenbrenner’s Ecological Systems Theory, they demonstrated that AI’s influence extends across multiple levels of the mental health system simultaneously, from the therapeutic alliance to broader cultural values and policy infrastructures. This systemic framing is particularly relevant to the action step, as it reminds counselors that responses to LLM-related breaches may need to extend beyond the immediate clinical encounter to address institutional, cultural, and policy-level dimensions.

We classify actions into two broad categories: actions directed to the client and actions directed at systemic levels. Each is addressed below distinctly, although realistically the categories may be interwoven. The course of action selected in a given case will depend on multiple factors, including applicable legal requirements, the specific circumstances of the case, the findings from Steps 1–3, and the counselor’s clinical judgment. While selecting a course of action, counselors should evaluate each option against relevant ethical principles and balance any competing values; for example, respecting client autonomy while fulfilling the obligation of non-maleficence.

### Actions directed to the client

Counselors should inform clients of the ethical concern in a manner appropriate to their digital literacy, emotional readiness, and details of their clinical case. When clients do not recognize that harm has occurred, such as in cases involving deceptive empathy or harmful AI-generated advice, counselors may provide psychoeducation regarding the limitations of LLMs and collaborate with the client to review concerning interactions, when clinically appropriate. A specific intervention is working with parents and school counselors to restrict or closely monitor LLM use for adolescents.

### Actions directed to systems

When an ethical breach involves an AI system used within an organization, counselors should report the concern through appropriate institutional channels. In cases involving harmful or discriminatory outputs, formal notification to the technology developer may also be warranted. As part of advocacy, counselors may choose to contact companies whose products operate at the intersection of AI and mental health, to raise awareness on behalf of clients. Counselors may also collaborate more directly with technology developers, such as by serving on ethics committees or advisory boards. Counselors may advocate for clients locally, regionally, nationally, or internationally, as well as drive change and reform in ethics, regulations, and law. Furthermore, counselors may advocate for AI education, infusing it into counselor training, supervision, and research (Association for Counselor Education and Supervision AI Task Force, 2026).

When LLM frameworks induce user dependency akin to a therapeutic relationship, practitioners should consider reporting these design and safety concerns to the developer. Counselors remain responsible for addressing concerns related to LLMs within the scope of their professional role, even when those concerns come from an AI system. Developers, organizations, and others involved may also share responsibility, but this model focuses specifically on the counselor’s ethical decisions and how they respond.

### Step 5: monitoring the consequences

Definition: This step involves ongoing evaluation of the outcomes following the action taken in Step 4. The counselor monitors the effects of their intervention on the client’s well-being, the therapeutic relationship, and any broader systemic implications. Monitoring is not a one-time event but a continuous process embedded within clinical practice. Drawing from Johnson et al.’s (2022) taxonomy, self-monitoring encompasses reviewing the decision and its effects, documenting the process, and re-engaging in ethical decision-making if needed. In the context of LLM use, monitoring is especially critical because the harms identified by Iftikhar et al. (2025), such as deceptive empathy, reinforcement of unhealthy beliefs, and crisis mismanagement, may persist, resurface, or evolve in new forms over time, even after an initial intervention has been implemented.

Counselors should evaluate whether the action taken resolved the ethical concern or whether residual harms remain. For example, if a client had developed an unhealthy attachment to an LLM, the counselor should assess whether that dependency has diminished following intervention or shifted in expression. If the client is still using the LLM, counselors should periodically revisit that use and reassess for emerging ethical violations across all five categories identified by Iftikhar et al. (2025).

Documentation and advocacy remain essential throughout this step. Counselors should maintain updated records that reflect changes in the client’s engagement with LLMs, the resolution status of the identified ethical breach, and any new concerns that emerge. When patterns of harm are observed across multiple clients or cases, counselors carry a professional responsibility to escalate their advocacy beyond the individual level by informing supervisors, contributing to institutional policy revisions, and engaging in professional discourse about the regulation of LLM use in therapeutic settings. As Johnson et al. (2022) note, the self-monitoring component of ethical decision-making includes reengaging in the process when needed, and in the context of rapidly evolving AI technologies, counselors should anticipate that the monitoring phase will frequently loop back into earlier steps of this model.

### Step 6: reflection and prevention

Definition: In alignment with the Johnson et al. (2022) taxonomy, intrapersonal reflection emphasizes self-awareness, prediction (anticipating downstream consequences), and when applicable, reengagement (returning to earlier parts of the decision-making process when new risks emerge). Counselors are encouraged to reflect on the case and beyond it, toward proactive safeguarding. Counselors should reflect on what factors, including their own biases, contributed to what occurred. This step pairs reflection with prevention; therefore, a major goal of reflection should be the development of concrete changes to reduce the likelihood of recurrence.

### Recommendations

Counselors should reflect on existing guardrails. For example, counselors should update informed consent procedures to address the fallibility of LLMs and AI. Counselors can also consider routine assessments of LLM use during intake. Prevention efforts should extend beyond the individual counselor. It is crucial to develop institutional safeguards, such as incorporating AI ethics into supervision and training, and developing clinic-level norms for addressing LLM use in treatment. Additionally, counselors have an advocacy role when systemic patterns emerge, particularly given the lag between technological change and formal ethical guidance. In such cases, prevention includes contributing to professional dialog, supporting policy development, and encouraging clearer standards for safe AI use in clinical settings.

Counselors should reflect on their awareness, knowledge, and skill at the intersection of cultural competence and LLM outputs, considering how LLMs may marginalize vulnerable communities. For example, adolescent clients may be especially vulnerable to digital attachment and influence. Consequently, counselors should evaluate existing safeguards for this population in particular. Also, they should remain updated on emerging literature about adolescent digital attachment and dependency, and suicide risk mediated by LLM and AI use.

The following illustration demonstrates how the model makes AI-specific ethical considerations explicit during the ethical decision-making process. It is followed by a case example that applies the six steps more fully to a client scenario.

## LLM-specific illustration

A client reports developing a strong emotional attachment to an LLM that they use for mental health support. A general ethical decision-making model could guide the counselor to define the problem, consider possible harms, consult ethical standards, select a response, and evaluate the outcome. These processes remain relevant. The present model adds considerations specific to the human–LLM context. It directs attention to simulated empathy, anthropomorphism, and digital attachment. The model considers responsibility across practitioners, clients, developers, and organizations. Also, it allows intervention to extend from the client encounter to concerns about the design and safeguards of the technology itself. During Step 1, the practitioner may explore the nature of the client’s relationship with the LLM, including whether the client interprets the system’s responses as evidence of genuine empathy. This is relevant because LLMs can produce anthropomorphic and seemingly empathic responses that may contribute to perceptions of emotional connection or dependency (Iftikhar et al., 2025). For Step 2, the practitioner could assess both potential risks and benefits rather than assuming that attachment itself constitutes harm. For example, the practitioner may consider whether the client is growing dependent on the LLM, as opposed to feeling empowered by it. They would also assess if the LLM is reinforcing unhealthy beliefs. For Steps 3 and 4, consultation and action may entail discussing these concerns with the client and developing a response that balances client autonomy and addressing the identified risks (to ensure non-maleficence). Rather than automatically recommending that the client stop using the LLM, the practitioner might work with their client to mutually establish boundaries around its use. They might also encourage additional human sources of support. Steps 5 and 6 would then require ongoing assessment of the client’s well-being. The practitioner would reflect on whether the response addressed the identified issues. The model does not encourage practitioners to assume that LLM use is inherently harmful. The purpose is to make AI-specific concerns (e.g., simulated empathy, emotional attachment) explicit within the ethical decision-making process.

## Hypothetical case example: Jenny

The following case is entirely hypothetical and is provided solely to illustrate application of our six-step model. It should not be interpreted as empirical evidence of the model’s effectiveness.

Jenny, a 22-year-old graduate student, started counseling presenting with anxiety, loneliness, and self-doubt following a breakup and relocation. She described using ChatGPT regularly for emotional support, especially at night, noting that it felt validating and “always available.” This raised concern with the counselor about how AI use might influence her thinking and relationships (Step 1). Some responses she received reinforced withdrawal (e.g., prioritizing distance from others) or overgeneralized beliefs (e.g., “people always leave”) (Step 2). The counselor approached this as an emerging, ambiguous concern, involving both potential benefit (emotional expression, accessibility) and risk (reinforcing maladaptive beliefs, reduced engagement with real-world supports) (Steps 1 and 2). After consulting with a colleague, the counselor framed the ethical issue as balancing respect for Jenny’s autonomy with the responsibility to monitor for harm and uphold non-maleficence (Steps 1 and 3).

The counselor considered multiple ways to address their concern, including psychoeducation or incorporating AI into treatment (Steps 3 and 4). The counselor ultimately took a collaborative, non-prescriptive approach, exploring what Jenny found helpful while offering transparent psychoeducation about how AI generates responses (Step 4). Using joint goal setting, they developed guidelines and checklists, such as noticing when AI use replaced human support, and underscoring the need to bring significant interactions into the session (Step 4). Over time, Jenny developed a more critical perspective about LLMs, describing the tool as “helpful sometimes, but not always accurate,” while occasionally using it during distress (Step 5). The counselor treated Jenny’s new perspective as clinically useful data, exploring what needs the AI was meeting and where counseling could be strengthened (Step 5). Throughout, the counselor remained mindful of AI’s imperfections and the possibility of harm (Step 2). The process required the counselor to confront their own preferences and biases and to remain open to multiple ethical interpretations rather than forcing a single outcome (Step 6). The counselor now also assesses their new clients’ usage of AI during intake and advocates for guidance for AI use from the institutional and professional level (Step 6).

## Discussion

In this article, we explained an ethical decision-making model to guide mental health professionals in addressing ethical challenges arising from the use of LLMs. The purpose of our article was to develop a model tailored to LLM-related ethical concerns by synthesizing established decision-making approaches and contemporary literature on the potential harms posed by LLMs. Subsequently, we developed a six-step, structured decision-making model. Building the model, we modified Johnson et al.’s (2022) taxonomy of ethical decision-making models, selecting procedural components most relevant to counseling and ethical dilemmas related to LLM use. Concurrently, we incorporated the findings of the Iftikhar et al. (2025) study, which identified ethical breaches commonly committed by LLMs when acting in a counselor role. We drew on other relevant research on ethical decision-making and LLM ethics, though these two studies served as our foundation.

Consistent with prior ethical decision-making research, our model retains several foundational procedural elements common across existing frameworks. For example, the problem recognition and interpretation step parallels components found in Forester-Miller and Davis (2016) and other widely used models. However, LLMs introduce ethical complexities that earlier frameworks were not designed to address. Our model therefore extends existing ethical decision-making approaches by incorporating AI-specific concerns such as deceptive empathy, algorithmic bias, digital attachment, and diffuse accountability structures. Also, our model departs from prior approaches by attending to the systemic and institutional dimensions of AI ethics.

Our model also builds on emerging scholarship in AI and mental health. For instance, Moore et al. (2025) identified the potential for LLMs to perpetuate mental health stigma, a concern reflected in our emphasis on cultural attunement and unfair discrimination as an explicit violation category. Also, Gonzalez and Dollarhide’s (2025) conceptualization of AI as a multi-systemic force informed our organization of “Step 4: Action” across client and advocacy domains. Our model, then, results from the synthesis of established decision-making models with current research on LLM risks.

### Limitations

Several limitations should be considered when interpreting our decision-making model. First, the proposed ethical decision-making model was developed primarily through the integration of two foundational sources: Iftikhar et al.’s (2025) empirical analysis of LLM-related ethical violations in counseling contexts and Johnson et al.’s (2022) taxonomy of ethical decision-making models. Although these sources provided a strong empirical and conceptual foundation, reliance on a limited number of core frameworks may have constrained the range of perspectives reflected in the model. Alternative taxonomies or research findings may have produced different emphases or organizational structures.

Second, the model is conceptual and has not yet been empirically validated. While grounded in existing scholarship and ethical decision-making literature, its practical utility and effectiveness in clinical settings remain unknown. Future research should evaluate whether mental health professionals find the model understandable and useful when navigating real-world ethical dilemmas involving LLMs.

Like most model-development studies, the model also reflects the interpretive judgments of the research team regarding the selection, organization, and prioritization of ethical concerns. We therefore encourage continued refinement, critique, and expansion of AI-specific ethical decision-making models as the technology and its applications continue to evolve. Finally, the rapid evolution of LLM technologies means that the ethical risks associated with AI are likely to change over time. Some concerns identified in our framework may diminish as systems develop, while new ethical challenges may emerge. Accordingly, this framework should be viewed as provisional rather than definitive.

### Implications

Despite these limitations, our model offers conceptual and practical implications. An important conceptual implication of our model is the recognition that LLMs introduce ethical challenges distinct enough to warrant dedicated decision-making models and frameworks rather than relying solely on existing models. This distinction has direct consequences for counselor training and supervision. Counselor education programs may consider incorporating LLM-specific ethical scenarios into curricula, which can help trainees navigate AI-related dilemmas before they encounter them in practice.

Another implication is that our model extends beyond procedural safeguards to address the relational dynamics sometimes present in human-to-LLM use. These include deceptive empathy, anthropomorphism, emotional dependency, and even attachment. Our model suggests that ethical evaluations of AI should extend beyond compliance concerns (e.g., data privacy) to examine how systems shape trust and attachment in counseling, as these may constitute the essence of an ethical dilemma. This relational framing challenges counselors to expand their ethical lens and be alert to the interpersonal dimensions of harm that LLM interactions may produce.

Our paper serves as a starting point for continued ethical-model construction. Other scholars may refine or challenge the model in response to new evidence and evolving technological capabilities. Thus, our model helps establish an intellectual foundation upon which future conceptual models or interdisciplinary collaborations can build. Finally, the model may assist organizations such as clinics and educational institutions by providing a structured and ethically grounded approach to ethical decision-making.

## Conclusion

This article introduces an ethical decision-making model to guide mental health professionals in addressing ethical challenges and dilemmas that may arise with the use of large language models (LLMs). Clients may use LLMs in a counselor-like capacity, and emerging research suggests that this practice raises a range of ethical concerns and potential harms. Our model provides a structured decision-making process to help mental health professionals respond to these situations. We do not present this model as definitive. The model is intended to evolve over time and to serve as one possible roadmap as LLM and AI technologies continue to advance.
